# Impacts of Environmental Factors on Pasting Properties of Cassava Flour Mediated by Its Macronutrients

**DOI:** 10.3389/fnut.2020.598960

**Published:** 2020-11-26

**Authors:** Yayuan Zhang, Lei Nie, Jian Sun, Yan Hong, Huabing Yan, Mingjuan Li, Xiangrong You, Ling Zhu, Fang Fang

**Affiliations:** ^1^Institute of Agro-Products Processing Science and Technology, Guangxi Academy of Agricultural Science, Nanning, China; ^2^Guangxi Key Laboratory of Fruits and Vegetables Storage-Processing Technology, Guangxi Academy of Agricultural Science, Nanning, China; ^3^Department of Statistics, Purdue University, West Lafayette, IN, United States; ^4^School of Food Science and Technology, Jiangnan University, Wuxi, China; ^5^Cash Crops Research Institute, Guangxi Academy of Agricultural Science, Nanning, China; ^6^Department of Food Science, Purdue University, West Lafayette, IN, United States

**Keywords:** cassava flour, component, pasting properties, environment, physicochemical traits

## Abstract

The impacts of environmental conditions on pasting and physicochemical properties were investigated using flour samples of the same cassava cultivar grown in seven different locations. Significant location differences in essential component (except for fiber) content of cassava flour were observed. Cassava flour showed obviously separated traits in the principal component analysis (PCA) of near-infrared spectra (NIR) according to geographical origins. The environmental effects were significant in the pasting properties of cassava flours. Sufficient precipitation and suitable low temperature promoted accumulation of starch in cassava, resulting in the high peak viscosity values of cassava flour. Pasting temperatures of cassava flour had a significant direct correlation with growth temperature and were negatively correlated with altitude. Precipitation from August to October showed a stronger direct correlation with trough and final viscosity. The results of this study indicated the possibility of predicting and controlling cassava flour quality and pasting properties according to the environmental conditions.

## Introduction

Cassava (*Manihot esculenta Crantz*) is known as the third most important source of calories after rice and maize throughout the tropical and subtropical regions (1) and recognized as one of the fastest expanding staple crops showing a continuously increased global production for 2008–2018 (238.5–277.1 million tons) (2, 3). Cassava can grow in infertile soils and has its own inherent tolerance to drought and salt stresses, together with its significant contribution to calorie per acre, which make it a promising crop to feed the growing global population (4–6).

Cassava roots have a short shelf-life of only 1–2 days after harvest because of the post-harvest physiological deterioration (7). One way to preserve cassava is by processing them into a dry product such as flour. Cassava flour produced from peeled cassava root has enormous potentials to replace wheat or maize-based flours for the manufacture of baking foods (8, 9). The use of cassava flours in the food industry as raw material is primarily governed by their composition and functional properties, which may be influenced by growth origins (10).

Starch is the most abundant component in cassava flour (about 74–85% of dry root weight basis) (11). The physicochemical properties of cassava starch have a major contribution to the texture and sensory attributes of cassava-based food products. Environmental conditions, such as growth temperature, rainfall, altitude have a large impact on the starch production and properties (12–15). According to Santisopasri et al. (12), when cassava crops were planted with initial water stress for the first 6 months, peak viscosity and swelling power of cassava starch were significantly higher, and its pasting temperature during gelatinization was significantly lower. However, the environmental temperature during growth period was not included into analysis. Gu et al. (16) reported that ambient temperature 3 months before cassava harvesting was positively correlated to paste clarity and pasting temperature but negatively correlated to freeze-thaw stability of cassava flour. Moreover, Karlström et al. (17) found that peak viscosity of cassava starch is statistically higher in plants grown in low altitude compared with those grown in intermediate altitude. These studies mainly focused on the effect of environmental factors on functional properties of starch. Nonetheless, the impacts of environmental conditions on functional properties of cassava flour being regulated by the cassava's main nutrients and characteristic of starch has not yet been thoroughly studied.

We hypothesize that the environment condition plays an important role on the essential composition and functional properties of cassava flours. In the currently study, the multilocational field experiments were conducted to assess the effect of environment on the main composition and resulting pasting properties of cassava flour from the same cultivar. The recently induced high-yield edible cassava cultivar, South China No. 12 (SC No. 12), was grown in seven different locations spread all over Guangxi Province (91,389 m^2^) locating in subtropics in China, the largest key producing region of cassava with northern latitude range of 20°54′-26°23′ and east longitude range of 104°29′-112°04′. The ambient temperature, precipitation, and altitude in these seven locations were recorded, and their correlations with the amount of key nutrients in cassava (i.e., starch, fiber, protein, and ash) as well as the characteristics of starch (i.e., granular size distribution and amylose content) that potentially influence its pasting properties were studied. With the information of the impacts of environmental conditions on cassava flour quality, it is possible to provide an opportunity for improving quality and functionality of cassava flours and minimizing the variation in flour quality from the cassava plants harvesting from different growth environments.

## Materials and Methods

### Plant Materials and Growth Conditions

The field experiment was carried out in seven different locations in Guangxi Province in China: Guilin (110°28′*E*, 25°29′*N*; 206 m.a.s.l.), Guiping (110°07′*E*, 23°38′*N*; 51 m.a.s.l.), Hepu (109°2′*E*, 21°33′*N*; 15 m.a.s.l.), Jingxi (106°41′*E*, 23°15′*N*; 781 m.a.s.l.), Leye (106°56′*E*, 24°78′*N*; 971 m.a.s.l.), Lingshan (109°29′E, 22°44′*N*; 71 m.a.s.l.), and Rongan (109°37′*E*, 24°24′*N*; 310 m.a.s.l.). South China No. 12 were planted from March 15 to March 20, 2015 and harvested from January 5 to January 6, 2016. Cassava plants (3 plants in Guilin and 5 plants in other locations) in the same geographical origin were selected at random for preparing the flours separately. Selected roots from the same cassava plant were peeled and washed, then chopped into pieces. The root slices were further dried at 45°C in a heat air cabinet drier for 8 h. Dried cassava samples were milled until all pass through 60 mesh sieves to obtain cassava flour for analysis. For each location, weather related data, including average daily temperatures and precipitations, were collected and calculated for each month from March to December 2015 throughout the whole cassava growth period. The general growth conditions are shown in Supplementary Table 1.

### Near-Infrared Spectroscopy

The NIR spectra of cassava flour samples were collected by DA7200 spectrometer (Perten Instruments Pty Ltd., Australia). Approximately 50 g of cassava flour was placed in a 7.5 cm diameter sample cell. Sample was scanned over the spectra range 950–1,650 nm with 2 nm interval in reflectance mode at 25°C. All acquisitions of the sample spectrum were performed in triplicate. Data form was recorded and converted to the absorbance.

### Composition Analysis

Starch, protein, fiber, and ash contents of cassava flours were measured using AOAC method (18). The amount of amylose was measured by iodine colorimetric reaction followed the method as described by Palav and Seetharaman (19).

### Particle Size Analysis

Particle size analysis of cassava starch granules was performed on aqueous dispersions of the isolated starches (100–200 mg) from cassava flours in alcohol (75% w/w, 1 mL). The particle size distribution was measured by laser diffraction particle size analyzer (Mastersizer 3000, Malvern Instruments Ltd., United Kingdom).

### Pasting Properties

Pasting properties of cassava flours (10% w/w dry basis) were analyzed using a Rapid Visco Analyzer (RVA-TecMaster, Perten Instruments Pty Ltd., Australia) and following AACC standard method 76-21.02 (20).

### Statistical Analysis

NIR spectra data were analyzed using Unscrambler software (version 9.8, CAMO ASA, Norway). The spectra were pre-processed by using the standard normal variate (SNV) followed by the Savitsky-Golay 1st order deviations. Then, the principal component analysis (PCA) was conducted using pre-processed data.

Explanatory variables were average daily temperatures and precipitation in each growth stage of cassava plant from March to December 2015.We defined the growth stage of cassava plant as the “seeding stage” (April to May), “tuber initiation stage” (June to July), “tuber bulking stage” (August to October), and “tuber maturity stage” (November to December). Pasting properties of cassava flour, which was prepared from harvested cassava roots were response variables. For each location, we averaged the data of each pasting property from different plants as the responses of the experiment. To measure the potential mediating variables, we combined all the cassava flour samples from the different plants in the same location and measured its composition, including starch, protein, ash, fiber and amylose content. We analyzed the average particle size distribution *D* [v, 50] (value of particle diameter at 50% in the cumulative distribution) as additional mediators. The data visualization and correlation analysis were generated using RStudio Version 1.1.414. Using the ggplot2_3.1.1 package1 (21) in RStudio, we generated explanatory variables vs. mediators and vs. response variables in each stage. To investigate whether there are linear correlations between these variables of interest, we later calculated the Pearson's correlation coefficients and the corresponding *P*-values using the ggpubr_0.2.1 package2 (22). A Pearson's correlation coefficient with a *P*-value smaller than 0.05 indicates a significant correlation between two variables.

## Results and Discussion

### Near-Infrared Analysis

**Figure 1:**
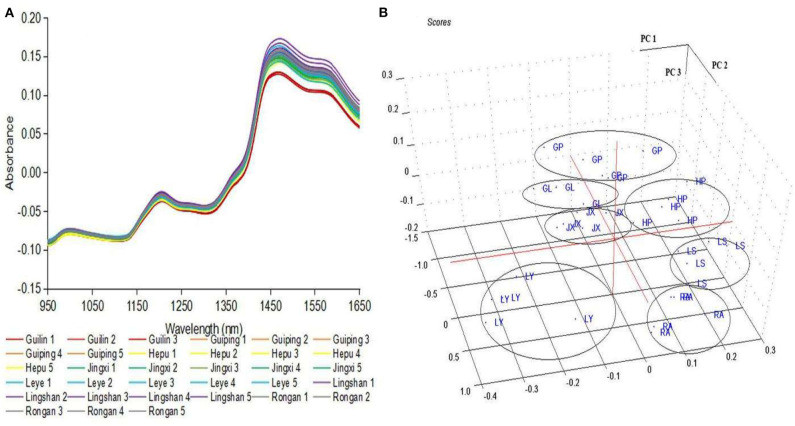
**(A)** The NIR spectral curves of all cassava flour samples. **(B)** Score plots from the principal component analysis of NIR spectral data of cassava flours. GL, Guilin; GP, Guiping; HP, Hepu; JX, Jingxi; LY, Leye; LS, Lingshan; RA, Rongan.

NIR spectra were characterized by absorption bands that were related to vibrations of different chemical bonds. As shown in Figure 1A, the NIR spectra of 33 cassava flours exhibited a broadly similar trend in peak and valley and displayed an obvious spectral peak at ~1,480 nm. Distinguishable spectral intensity differences at a certain wavelength were observed among 33 samples that originated from their differences in the content of compositions. However, it is difficult to obtain sample composite information directly from the spectra. Thus, there is a need for data processing methods that transform the measured spectral data into the sample properties of interest (23).

PCA was performed to evaluate an overview of the similarities and differences among the cassava flours from different geographical origins. Figure 1B showed the score plots corresponding to the first three principal components. The first three PCs accounted for 97% of the total variance in the spectral data set (91% the first, 4% the second, and 2% the third). Two closer spots on the score plot generally indicate a higher degree of similarity compared to the spots farther apart. A separation existed between the cassava flours according to the geographical origin. Replicate samples of the cassava flours from the same location can be grouped in the same cluster. There was no obvious overlap among the seven geographical origins. It was observed that cassava flours from Leye showed negative loading on PC1 and PC2 of the score plot. It is at some distance from the other spots. Cassava flours from Guilin, Jingxi, Rongan, and Guiping were located close to zero in the PC1. Some plots presenting Guiping and Rongan were distributed close to the positive direction of PC1. Cassava flours from Hepu and Lingshan can be observed on the right-hand side of the score plot.

It is worth noting that the spatial distribution of the samples associated with PC1 is highly correlated with average temperature. Locations along PC1 toward positive direction tend to have a higher average daily temperature. The data points representing Leye (denoted by LY), a city having the lowest average daily temperature (20.0°C), gathered toward the negative direction of abscissa. The average temperature of Guilin (denoted by GL, 22.4°C) and Jingxi (denoted by JX, 22.5°C) during the trial were very close (Supplementary Table 1). Samples from these two cities were grouped toward ordinate and can't be separated based on the PC1 scores. Guiping (denoted by GP, 24.5°C) and Rongan (denoted by RA, 24.3°C) had a slightly higher temperature than the other locations and their data points were distributed toward a more positive direction of PC 1. All the replicated samples from Hepu (denoted by HP, 26.2°C) and Lingshan (denoted by LS, 24.8°C), cities having higher temperatures than Guiping, located in the positive direction of PC1. Therefore, it was speculated that ambient temperature plays a particularly important role on the essential composition of cassava flours.

### Proximate Composition and Starch Characterization of Cassava Flours

The PCA of NIRs indicated that the overall nutrient compositions were different in cassava flour from different geographical origins. We further measured the contents of main nutrients in cassava flour, including starch, protein, fiber, and ash (mainly minerals), as given in Table 1. Starch is the most abundant ingredient in cassava and its content in the cassava flours from the seven different locations ranged from 75.35 to 82.0% (dry basis). These values were in agreement with those reported by Aryee et al. (67.92–88.11%) (24) and Charoenkul et al. (80–86%) (25). Cassava flour from Guilin (110°28′*E*; 25°29′*N*, the northernmost of the seven locations) had the highest starch content, while flour from Hepu (109°2′*E*; 21°33′*N*, the southernmost of the seven locations) had the lowest value. Amylose contents of cassava flours ranged between 15.0 and 17.35% with small variations (coefficient of variation ~6.1%) as given in Table 1. Cassava flours from Hepu (109°2′*E*; 21°33′*N*) and Leye (106°56′*E*; 24°78′*N*) had significant lower amylose content (*p* < 0.05) as compared with cassava flours from other five locations.

**Table 1:** Proximate composition contents in cassava flour and particle size of cassava starch.

	**Starch**	**Protein**	**Fiber**	**Ash**	**Amylose**	**Particle size**
**Cities**	**(%)**	**(%)**	**(%)**	**(%)**	**(%)**	(μm)^*^
Guilin	82.0 ± 1.2^a^	1.9 ± 0.2^c^	2.8 ± 0.3^a^	2.4 ± 0.4^cd^	17.2 ± 0.4^a^	9.0 ± 0.2^d^
Guiping	78.1 ± 0.8^bc^	3.0 ± 0.5^bc^	2.0 ± 0.5^a^	1.9 ± 0.2^d^	17.1 ± 0.4^a^	10.2 ± 0.1^bc^
Hepu	75.35 ± 0.6^c^	4.3 ± 0.4^a^	2.4 ± 0.1^a^	3.9 ± 0.2^a^	15.1 ± 0.2^b^	10.1 ± 0.0^bc^
Jingxi	79.3 ± 1.4^ab^	2.5 ± 0.4^bc^	2.5 ± 0.3^a^	2.0 ± 0.5^d^	16.6 ± 0.4^a^	10.0 ± 0.1^c^
Leye	78.6 ± 1.7^abc^	2.6 ± 0.3^bc^	2.3 ± 0.1^a^	3.6 ± 0.1^ab^	15.0 ± 0.1^b^	10.1 ± 0.1^bc^
Lingshan	77.8 ± 0.9^bc^	2.1 ± 0.7^bc^	2.5 ± 0.2^a^	3.1 ± 0.3^bc^	17.4 ± 0.6^a^	10.5 ± 0.2^a^
Rongan	77.5 ± 1.6^bc^	3.6 ± 0.6^ab^	2.7 ± 0.5^a^	2.9 ± 0.4^bc^	16.9 ± 0.1^a^	10.3 ± 0.3^ab^
Mean	78.4	2.9	2.5	2.8	16.5	10.0
CV (%)	2.6	29.2	10.6	28.0	6.1	4.7

* *Particle size: D [v, 0.5]*.

*CV, Coefficient of Variation*.

*Data (mean ± standard deviation) in the same column with different lowercase letters are significantly different (p < 0.05)*.

The content of protein in cassava flours was in a range of 1.9–4.25% with large variations (coefficient of variation ~29.2%). Cassava flours from Hepu had the highest protein content. The mean protein content of cassava harvested from all seven locations (2.86%) were slightly lower when compared to previously reported data (3.06%) (26). Ceballos et al. (26) suggested that cassava from Asia tended to have lower-than-average protein levels. In this study, protein content was inversely correlated with starch content (*p* < 0.05, Table 3). Fiber contents of cassava flours did not vary significantly giving a range of 2.0–2.75%.

Ash content represented the total amount of mineral and inorganic materials in cassava flour. The total ash content in cassava flour was largely varied among different growth environments (1.9–3.9%, dry cassava flour basis). An inverse correlation was found between ash and amylose content in the cassava flours (*p* < 0.05). There were significant differences between the median diameter (*D* [v, 0.5]) levels of the cassava starch from different geographical origins. The values ranged between 9.0 and 10.5 μm. Particle size (*D* [v, 0.5]) showed a negative correlation with the starch content (*p* < 0.05).

### Pasting Properties of Cassava Flour

Pasting properties of flour play an important role in determining the cooking and baking qualities (27). The pasting profiles of cassava flours are shown in Table 2. Pasting temperature gave an indication of the minimum temperature required for flour cooking. It was observed that pasting temperature of cassava flour from different geographical origins varied significantly (*p* < 0.05) ranging from 63.1°C (Leye) to 71.3°C (Hepu) with negligible differences among replicated samples.

**Table 2:** Pasting properties of cassava flours harvested from different locations.

	**PV**	**Trough**	**BD**	**FV**	**SB**	**PT**	**PP**
**Cities**	**(mPa·s)**	**(mPa·s)**	**(mPa·s)**	**(mPa·s)**	**(mPa·s)**	**(min)**	**(^**o**^C)**
Guilin	4994 ± 239^a^	1651 ± 25^b^	3343 ± 216^a^	2407 ± 18^ab^	756 ± 34^a^	5.3 ± 0.1^cd^	65.9 ± 0.5^c^
Guiping	4571 ± 259^b^	1602 ± 98^b^	2969 ± 194^b^	2270 ± 95^bc^	667 ± 35^abc^	5.7 ± 0.2^bc^	68.3 ± 0.6^b^
Hepu	3954 ± 133^c^	1646 ± 89^b^	2309 ± 154^c^	2403 ± 63^ab^	703 ± 59^abc^	6.3 ± 0.5^a^	71.3 ± 0.0^a^
Jingxi	5139 ± 73^a^	1666 ± 46^b^	3473 ± 70^a^	2395 ± 53^ab^	729 ± 35^ab^	5.0 ± 0.1^d^	65.2 ± 0.5^c^
Leye	4474 ± 307^b^	1871 ± 84^a^	2603 ± 226^c^	2496 ± 98^a^	625 ± 29^c^	6.5 ± 0.4^a^	63.1 ± 0.5^d^
Lingshan	3855 ± 87^c^	1660 ± 90^b^	2295 ± 78^c^	2309 ± 57^bc^	648 ± 45^bc^	5.9 ± 0.1^ab^	70.6 ± 0.7^a^
Rongan	4053 ± 241^c^	1561 ± 35^b^	2493 ± 217^c^	2253 ± 35^c^	692 ± 50^abc^	5.7 ± 0.1^bc^	67.7 ± 0.2^b^
Mean	4434	1665	2783	2357	689	5.8	67.4
CV (%)	11.4	5.9	17.4	3.7	6.6	9.0	4.4

*PV, peak viscosity; BD, breakdown; FV, final viscosity; SB, setback; PT, peak time; PP, pasting temperature; CV, Coefficient of Variation*.

*Data (mean ± standard deviation) in the same column with different lowercase letters are significantly different (p < 0.05)*.

Peak viscosity of cassava flour shows the ability of starch to swell freely before their physical breakdown, which is strongly correlated with final product quality (28, 29). With applied a constant shear (160 rpm) at a high temperature (95°C), swollen starch granules disrupt to small fragments then further break into dispersed molecules, a process resulting in a decrease in viscosity which referred to “breakdown” viscosity in the pasting profile. Peak viscosity and breakdown of cassava flours ranged from 3855 to 5139 mPa·s and 2295 to 3473mPa·s, respectively (Table 2). Pearson correlation analysis showed that peak viscosity was strongly positively correlated with breakdown values (*p* < 0.01, Table 3). Significantly higher peak viscosity and breakdown were observed for cassava flour from Guilin and Jingxi as compared to other five locations. The peak viscosity and breakdown values varied greatly among the replicate samples, indicating a great variation from plant to plant from the same location. Peak time is the time at which viscosity reaches the peak value. Flours with short peak time were suggested to have low resistance to swell and rupture (30). Flours from Hepu had the longest peak time, and flours from Jingxi had the shortest. Peak time was negatively correlated with the breakdown value (*p* < 0.05).

**Table 3:** Correlation coefficient between weather conditions with mediating factors (starch, protein, fiber, and ash contents, amylose content, starch particle size) and response variables (pasting properties).

	**PRE**	**TEMP**	**ALT**	**Starch**	**Protein**	**Fiber**	**Ash**	**AMY**	**Particle**	**PV**	**Trough**	**BD**	**FV**	**SB**	**PT**	**PP**
PRE	1															
TEMP	−0.13	1														
ALT	−0.22	−0.86^**^	1													
Starch	0.67	−0.62	0.27	1												
Protein	−0.27	0.57	−0.26	−0.83^*^	1											
Fiber	0.61	−0.05	0.04	0.33	−0.13	1										
Ash	−0.27	0.08	0.03	−0.57	0.42	0.1	1									
AMY	0.4	0.22	−0.42	0.46	−0.49	0.2	−0.74^*^	1								
Particle	−0.83^*^	0.34	−0.01	−0.77^*^	0.4	−0.45	0.25	−0.13	1							
PV	0.34	−0.59	0.47	0.78^*^	−0.51	0.06	−0.68	0.17	−0.65	1						
Trough	−0.31	−0.76^*^	0.72^*^	0.13	−0.3	−0.22	0.45	−0.65	−0.04	0.15	1					
BD	0.4	−0.44	0.32	0.78^*^	−0.5	0.11	−0.79^*^	0.34	−0.64	0.98^**^	−0.05	1				
FV	−0.06	−0.64	0.62	0.23	−0.2	0.05	0.42	−0.71^*^	−0.4	0.35	0.85^**^	0.18	1			
SB	0.64	0.13	−0.18	0.45	−0.05	0.6	−0.43	0.31	−0.72^*^	0.55	−0.49	0.64	0.01	1		
PT	−0.4	0.03	0	−0.61	0.42	−0.36	0.86^**^	−0.71^*^	0.4	−0.68	0.51	−0.81^*^	0.29	−0.73^*^	1	
PP	−0.19	0.96^**^	−0.87^**^	−0.63	0.45	−0.08	0.23	0.18	0.37	−0.7	−0.6	−0.58	−0.55	−0.01	0.18	1

*PRE, precipitation was collected from April to December, 2015; TEMP, average daily temperature was collected and calculated from April to December, 2015; ALT, altitude; Amylose, amylase content in cassava flour; Particle: D [v, 0.5]; PV, peak viscosity; BD, breakdown; FV, final viscosity; SB, setback; PT, peak time; PP, pasting temperature*.

* *Significant difference at p < 0.05*.

** *Significant difference at p < 0.01*.

Trough viscosity reflects the hot paste viscosity of flour and was reported to be important in cake batter setting during baking (31). The result showed that cassava flours from Leye had significant higher trough viscosity (*p* < 0.05) as compared with other cassava flours (Table 3). There were no significant differences in trough viscosity among the cassava flours from the rest of six locations. Setback indicated a tendency of starch retrogradation that has an inverse relationship with stability/shelf-life of starchy food products (31). A low setback value indicates that flour can be used for products where high starch stability is required. In comparison to the rest of other samples, cassava flour from Guilin showed the highest setback, while cassava flour from Leye had the lowest. Final viscosity represents the stability of cooked pastes, which is often correlated with final product quality (32). Final viscosity and setback values of cassava did not have large variations among different growth locations. The stronger positive correlation between final viscosity and trough viscosity was observed (*p* < 0.01).

The correlations between pasting properties of cassava flour and their potential mediating factors were analyzed. Peak viscosity and breakdown values were positively correlated with starch content. Similar result was also reported by Charoenkul et al. (25), who suggested that flours containing higher starch content displayed higher paste viscosities. Ash content inversely correlated with breakdown but directly correlated with peak time, indicated that the mineral and inorganic compositions in cassava flour may impede starch granule swelling. Cassava flour contained 7–15 mg phosphorus and around 10–21 mg calcium per 20.8–28.7 g dry matter (33), which most likely existed in ionic forms. The impact of ash on pasting properties may act as a direct effect of inorganic salts existing in ashes and/or an indirect effect of ashes through interaction with other compositions in cassava flours (34). A negative correlation was observed between setback and starch particle size (*D* [v, 0.5]). Amylose content was indirectly correlated with final viscosity and peak time. In addition, protein and fiber contents had no significant correlation with all responses.

### Impacts of Environmental Factors on Pasting Properties of Cassava Flour

**Figure 2:**
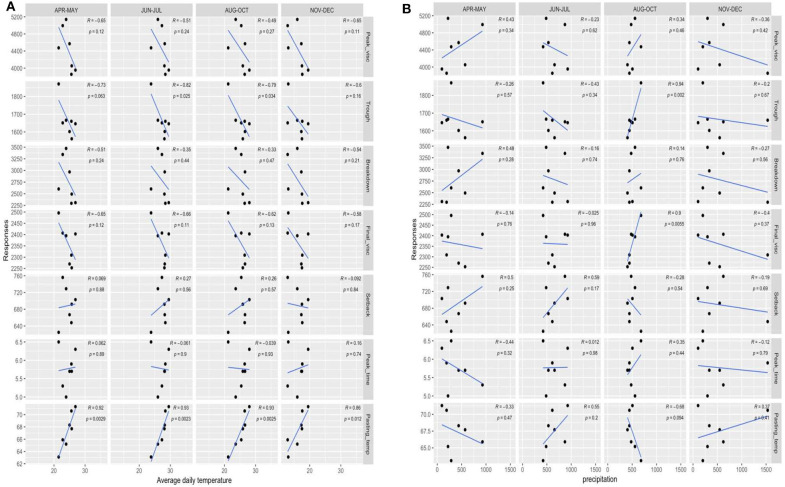
**(A)** Correlation between average daily temperature and responses. **(B)** Correlation between precipitation and responses. Peak_visc, peak viscosity; Final_visc, final viscosity; Pasting_temp, pasting temperature; Starch_cont, starch content in cassava flour; Fiber_cont, fiber content in cassava flour; Protein_cont, protein content in cassava flour; Ash_cont, ash content in cassava flour; AMY_cont1, amylose content in cassava flour; Particle_size, D [v, 0.5].

In order to understand the correlations between environmental factors and pasting properties of cassava flour, we further plotted average daily temperatures, precipitation, and altitude vs. response variables (i.e., the pasting properties of cassava flour). Pasting temperatures had significantly direct correlation with average daily temperature value in general over the whole cassava growth period (Figure 2A) and were negative correlated with altitude (*p* < 0.01). In these seven locations, the average temperature showed a negative correlation with altitude. Pasting temperature refers to the temperature that when initial onset of viscosity occurs and is mainly related to initial swelling of starch. When using cassava flour as partial substitute to wheat flour in baking products, high quality of bread products associates with minimized difference in pasting properties between cassava flour and wheat flour (28). For example, it has been reported that starch gelatinization in wheat flour initiates within the range of 58–64°C (28). Therefore, cassava flour produced from low-temperature and high-altitude zone would be a more promising material as a partial substitute to wheat flour for baking.

Trough viscosity was positively correlated with altitude and negatively correlated with average temperature (*p* < 0.05). The direct correlations between trough viscosity and average daily temperature was observed in tuber initiation stage (June–July) and tuber bulking stage (August–October). In addition, precipitation from August to October (tuber bulking stage) showed a strong direct correlation with trough and final viscosity (*p* < 0.01). An adequate amount of rainfall during the tuber bulking stage of cassava growth contributed to increase trough and final viscosities of cassava flour (Figure 2B). It was reported by Karlström et al. (17) that peak viscosity of cassava starch is higher in plants grown in intermediate altitude locations than those grown in low altitudes. In this study, the cassava flours from intermediate altitude zones (Jingxi and Leye) had higher average peak viscosities than those from low altitude zones (Guiping, Hepu, and Lingshan), while no statistical correlation was found.

### Impacts of Environmental Factors on Potential Mediating Factors

**Figure 3:**
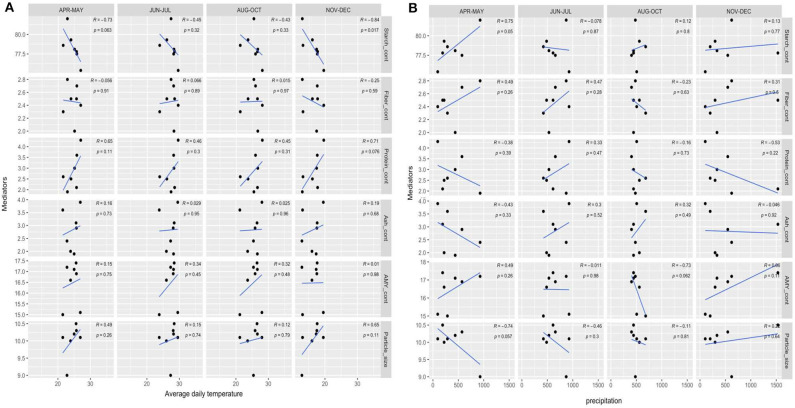
**(A)** Correlation between average daily temperature and potential mediators. **(B)** Correlation between precipitation and potential mediators. Peak_visc, peak viscosity; Final_visc, final viscosity; Pasting_temp, pasting temperature; Starch_cont, starch content in cassava flour; Fiber_cont, fiber content in cassava flour; Protein_cont, protein content in cassava flour; Ash_cont, ash content in cassavaflour; AMY_cont1, amylose content in cassava flour; Particle_size, D [v, 0.5].

Total starch content had an inverse correlation with average daily temperature from November to December (tuber maturity stage) (Figure 3A). Although there was no significant correlation between starch content in cassava flour and average daily temperature over the whole cassava growth period (Table 3), it was observed that cassavas grown in low-temperature zones tended to have higher starch content than that in high-temperature zones (Table 1 and Supplementary Table 1). Hot and cold temperature on starch biosynthesis of some crops (e.g., wheat) has been well studied, although it remains unclear in cassava. As proposed by Fang et al. (35), high ambient temperature reduced activity of key enzymes and their genes expression associated with starch biosynthesis. Therefore, the low ambient temperature, especially during the tuber maturity stage may yield high starch content that contributes to high peak viscosity and breakdown value in cassava flour.

As shown in Figure 3B, precipitation from April to May (seeding stage) had a direct correlation with the starch content and an indirect correlation with amylose content (*p* < 0.05), indicating sufficient precipitation promoted the accumulation of starch in cassava, which may contribute to high peak viscosity and breakdown. This result was in agreement with the previous findings showing that the cassava receive sufficient water especially in the initial stage will lead to dramatic gains in cassava starch content (12). As proposed by Santisopasri et al. (12), rainfall has a direct or indirect impact on starch quality, expressed by soil temperature, nutrient transportation, air humidity and light intensity. Initial water stress may allow the cassava plant to enter or remain in a dormant state. So, it can retard the normal growth and development of plants. The average particle size *D* [v, 0.5] was in inverse correlation with the precipitation from April to May (seeding stage). High precipitation in the early growth stage of cassava may increase the setback value of cassava flour by mediating particle size.

## Conclusions

The impacts of environmental conditions (ambient temperature, precipitation, and altitude) on the properties of cassava flour were assessed in this study. The NIR spectra of cassava flours from seven different locations could be distinguished by principal component analysis (PCA), and spatial distribution of the sample spots associated with PC1 was highly correlated with average daily temperature during growth period. Ambient temperature had a strong positive impact on the pasting temperature of cassava flour throughout the whole growth period and had a negative impact on its trough and final viscosities during the tuber initiation stage and tuber bulking stage. Suitable low temperature and adequate amount of precipitation could contribute to high peak viscosity by mediating starch content accumulation in cassava flour. By statistical analysis, the fiber content of cassava flour was not affected by planting location. Protein, fiber, and ash contents had no significant correlation with the investigated environmental factors (ambient temperature, precipitation, and altitude). Soil environment and genotypes may be major sources of variation for these compounds. The mechanism of environmental conditions influencing the pasting properties of cassava flour via mediating factors (starch, fiber, protein, and ash contents, amylose content, and starch particle size) was complex. The results of this study indicated the possibility of predicting cassava flour quality and pasting properties from the environment conditions of different cassava growth stage and deepened the understanding of relationship between pasting properties of cassava flour and its mediating factors (i.e., proximate composition contents and characteristics). Further studies can investigate the influence of other environmental factors such as soil and a larger sample size from different genotypes to create predictive models.

## Data Availability Statement

The original contributions presented in the study are included in the article/Supplementary Materials, further inquiries can be directed to the corresponding authors.

## Author Contributions

YZ: conceptualization, formal analysis, investigation, methodology, writing—original draft, review, editing, supervision, and funding acquisition. LN: formal analysis and visulization. JS, YH, and XY: data collection. BY: resources. LZ: data analysis. ML: methodology and resources. FF: conceptualization, methodology, formal analysis, writing—original draft, review, and editing. All authors contributed to the article and approved the submitted version.

## Conflict of Interest

The authors declare that the research was conducted in the absence of any commercial or financial relationships that could be construed as a potential conflict of interest.
