# Supplementary material for: Impacts of Environmental Factors on Pasting Properties of Cassava Flour Mediated by Its Macronutrients
Source: Front Nutr. 2020 Nov 26;7:598960. doi: 10.3389/fnut.2020.598960 (PMC7725801; doi:10.3389/fnut.2020.598960)
Supplement: Supplementary file 1 [file Table_1.DOCX]

Supplementary Material

**Supplementary Table** S1. The general growth conditions of cassava.

| Cities | Longitude | Latitude | Altitude (m.a.s.l.) | Average daily temperature^a^ (^o^C) | Total precipitation^b^ (mm) |
| --- | --- | --- | --- | --- | --- |
| Guilin | 110°28’*E* | 25°29’*N* | 206 | 22.4 | 2901.1 |
| Guiping | 110°07’*E* | 23°38’*N* | 51 | 24.5 | 1747.6 |
| Hepu | 109°2’*E* | 21°33’*N* | 15 | 26.2 | 1631.6 |
| Jingxi | 106°41’*E* | 23°15’*N* | 781 | 22.5 | 1561.4 |
| Leye | 106°56’*E* | 24°78’*N* | 971 | 20.0 | 1591.4 |
| Lingshan | 109°29’*E* | 22°44’*N* | 71 | 24.8 | 1531.9 |
| Rongan | 109°37’*E* | 24°24’*N* | 310 | 24.3 | 2179.9 |

^a^Average daily temperatures were collected and calculated from April to December 2015.

^b^Total precipitation was collected from April to December 2015.
